# Impacts of Natural Hazards on Primary Health Care Facilities of Iran: A 10-Year Retrospective Survey 

**DOI:** 10.1371/currents.dis.ccdbd870f5d1697e4edee5eda12c5ae6

**Published:** 2013-06-28

**Authors:** Ali Ardalan, Hani Mowafi, Homa Yousefi

**Affiliations:** Department of Disaster & Emergency Health, National Institute of Health Research, Tehran University of Medical Sciences, Tehran, Iran; Department of Disaster Public Health, School of Public Health, Tehran University of Medical Sciences, Tehran, Iran; Boston University; Ministry of Health & Medical Education, I.R.IranDisaster Management & Risk Reduction Unit at the Deputy for Public Health

## Abstract

Public health facilities in Iran are exposed to a wide range of natural hazards. This article presents the first survey of the impacts of such natural hazards on primary health care (PHC) centers in Iran from 2001 to 2011. A retrospective survey was conducted in 25 out of 30 provinces of Iran. Archival reports at provincial public health departments were cross-referenced with key informant interviews. During a 10-year period, 119 natural hazard events were recorded that led to physical damage and/or functional failure in 1,401 health centers, 127 deaths and injury or illness in 644 health staff. Earthquakes accounted for the most physical damage and all health-worker deaths. However, there was an increasing trend of impacts due to hydro-meteorological hazards. Iran’s health system needs to establish a registry to track the impact of natural hazards on health facilities, conduct regular hazard and vulnerability assessments and increase mitigation and preparedness measures. Keywords: Disaster, primary health care, facility, Iran, natural hazard Corresponding author: Ali Ardalan MD, PhD. Iran’s National Institute of Health Research, Tehran University of Medical Sciences. Harvard Humanitarian Initiative. Email: aardalan@tums.ac.ir

## Introduction

Natural disasters adversely affect the health of populations directly through injury and death; increased physical and mental illness; displacement and disruption of social networks; as well as destruction of physical surroundings and personal property 1. Damage to the structure and function of health facilities can have further impacts by impairing the ability of communities to respond to these calamitous events.

There are limited data of the impact of natural hazards on primary health centers. What data does exist largely focuses on hospitals. There are some unstructured data on damages included in regional WHO reports. For example, according to PAHO 2 from 1981 to 1996 there are records of 93 hospital and 538 health centers that suffered damage in Latin America and the Caribbean; 52 health facilities were destroyed in the Jammu and Kashmir earthquake of 2005; 49 health facilities sustained damage in the Indonesian Floods in 2007; and 322 hospitals and health centers were damaged in the South-Asia tsunami of 2004 3. Data on the destruction of health facilities remains sparse but there is increasing interest in its collection as governments and the world health community take account of the impacts of natural disasters on health systems. While the 2008-2009 Global Campaign of Hospitals Safe from Disasters 4 emphasized the vital role of primary health care facilities in disasters, no scientific publications to date have analyzed the impact of natural hazards on such facilities.

Over the last two decades, the Islamic Republic of Iran (I.R. Iran) has established and strengthened an extensive network of primary health care (PHC) system comprised of a multi-tiered system that spans both urban and rural areas. The effectiveness of this network has been measured in terms of declining child and maternal mortality rates, prevention of communicable diseases, and increased public health awareness 5 ^,^ 6. The PHC centers are also formally the key nodes for delivery of public health services in times of disaster. Even in the case of the devastating 2003 Bam earthquake, where more than 90% of health facilities were destroyed, all dispatched health teams from other provinces organized their field centers and service plans along the PHC network guidelines. The same has held true for more recent major disasters including the Zarand earthquake (2005), Lorestan earthquake (2006), cyclone Gonu (2007) and the East Azerbaijan earthquake (2012).

Despite the key function of PHC facilities in Iran’s disaster plan, there was no aggregated data about the past impacts of disasters on the structure and function of these centers. Such information is essential for effective policy making, disaster planning and resource allocation in the future. This article presents findings of a retrospective impact assessment of natural hazards on primary health centers of I.R. Iran during a 10-year period from 2001 to 2011.

## Methods

This retrospective survey was carried out from April to June 2012 covering 42 public health departments in all 30 provinces of I.R. Iran. The goal was to record all natural hazard events from 2001 - 2011 that had an adverse impact on health centers of the PHC network and their staff. Responses were received from 36 of 42 (85.7%) public health departments (PHD) representing 83.5% of primary healthcare centers (PHC) in areas that experienced 7 81.5% of the natural hazard events occurring in Iran during the relevant period 8 (figure 1).

**Description of respondent and non-respondent public health departments (PHD) in terms of number of primary health care (PHC) centers and natural hazard events in corresponding areas during the study period:**
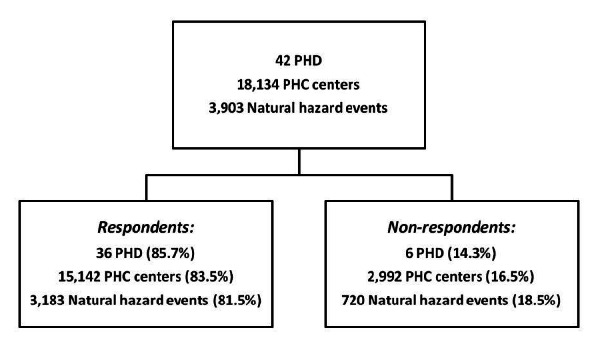

Impact was defined across four domains – personnel, structural damage, non-structural damage and functional failure. The degree of impact was further categorized as mild, moderate or severe. As this was a retrospective study, the designations of severity were recorded as they had been contemporaneously recorded in PHC records. In instances where damages were expressed in percentages, data collectors were asked to categorize the damages using the following guideline - <30% was considered mild; between 30-70% was moderate; and >70% was severe.

The data were further categorized by the type of PHC facility affected including: health house (HH) that is in charge of primary health service delivery in one or more villages; rural health center (RHC) that is responsible for management of several HHs; health post (HP) that delivers services in suburban areas; urban health center (UHC); district health network center (DHNC) – an administrative office that supervises the district hospital as well as the district public health center (DPHC) which is the administrative head of the smaller facilities. Measured impacts on personnel included number of staff directly killed as part of a hazard event, as well as injuries or illnesses that resulted from a hazard event. Structural damage was defined as a damage to wall, roof or columns of the building. Non-structural damage included damage to equipment, architectural elements or critical utilities (water, electricity, natural gas). Functional failure was defined as any reduction in the delivery of health services as a result of the hazard occurrence. Damage to the structure and function of hospitals was not assessed as it was explicitly outside the scope of this study.

Each public health department (PHD) has a disaster management and risk reduction unit and the coordinator for this unit served as the local focal point for data collection and reporting. Since no registry system existed at the PHD level to record the impact of natural disasters on PHC facilities, a two-step iterative approach was used to collect the data. The first source was the archive of reports of the local unit of health network expansion (UHNE). This unit is present in each PHD office and is primarily charged with management of PHC facilities. In addition, key informants including head and senior staff of UHNE were interviewed to capture any documents not initially obtained from the archival review.

A data collection form was developed and validated for face and content validity by experts in health systems and disaster management. The form collected the characteristics of each PHD, as well as the hazards and impacts related to those hazards that occurred over the relevant period. The form was composed of closed-ended questions and was written in Farsi. The form was also piloted in three provinces. A translation of the data collection form is attached (Appendix 1). The data collection team received training on the study objectives, data collection methods and the instrument used. The data collectors were instructed to record the type of hazards as they were noted in the PHDs documents so that the data collectors did not interpret the types of events. During the survey period, one of the principal investigators was available during working hours to answer queries from the data collection team.

Thirty-six out of 42 PHDs participated in the survey covering 25 provinces. Thirty of the 36 PHDs reported at least one significant hazard event. If an event affected more than one province, it was considered as a single event in aggregate analysis. For the purpose of mapping at the provincial level such hazards were analyzed separately in each province. Data were reported on a weekly basis and analyzed regularly. In the case of missing or outlier data, field collection teams were sent back for clarification. Descriptive indices were calculated using SPSS 16.0 and displayed using Microsoft Excel (2007).

This study was reviewed by the relevant department of Iran’s Ministry of Health and Medical Education and considered exempt as no human subjects research was conducted and no sensitive data was collected.

## Results

From 2001 to 2011, 119 natural hazard events were recorded in 25 provinces of Iran (11.9 hazards per year) that affected primary health care centers and threatened the lives and safety of health staff. This represents 3.1% of the 3,903 natural hazard events overall occurring in the same time period in Iran 8. These 119 events led to physical damage or functional failure of 1,401 health centers, injury or illness of 644 and death of 127 health workers. Kerman, Sistan and Balouchestan and Lorestan were the provinces with the highest adverse impacts on health centers from natural hazards (figure 2).

**Provincial distribution of affected primary health care centers by natural hazards, I.R.Iran, 2001-2011:**
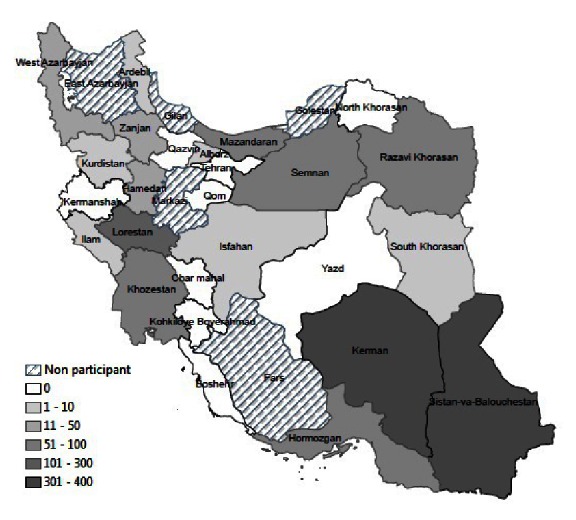

Earthquakes accounted for the most physical damage and functional failure of primary health centers in Iran. In addition, they accounted for all health-worker deaths and three-quarters of cases of illness and injury (table 1). An increasing trend of natural hazards’ occurrence was observed over the ten-year period of the study, especially due to hydro-meteorological hazards, with widespread adverse impact on the PHC system (figure 3). No deaths were reported due to hydro-meteorological hazards.

**Table 1: Frequency and impacts of natural hazards on primary health care system by type of hazards, I.R.Iran, 2001-2011:** 

Hazard type	Hazard frequency	Affected health staff		Affected health facilities		
		Death	Injury/ illness	Structural damage	Non-structural damage	Functional failure
	n (%)	n (%)	n (%)	n (%)	n (%)	n (%)
Earthquake	32 (26.9)	127 (100)	500 (77.6)	375 (53.8)	534 (63.4)	892 (64.7)
Landslide	28 (23.5)	0	0	24 (3.4)	25 (3.0)	13 (0.9)
Subsidence	15 (12.6)	0	4 (0.6)	18 (2.6)	4 (0.5)	2 (0.1)
Storm	13 (10.9)	0	105 (16.3)	109 (15.6)	100 (11.9)	252 (18.3)
Torrential rain	10 (8.4)	0	0	60 (8.6)	17 (2.0)	48 (3.5)
Flood	10 (8.4)	0	0	48 (6.9)	46 (5.5)	9 (0.7)
Extreme Weather	8 (6.7)	0	25 (3.9)	57 (8.2)	110 (13.1)	78 (5.7)
Dust/Sand Storm	3 (2.5)	0	10 (1.6)	6 (0.9)	6 (0.7)	84 (6.1)
**Total**	**119 (100)**	**127 (100)**	**644 (100)**	**697 (100)**	**842 (100)**	**1,378 (100)**

**Occurence of natural hazards with an impact on primary health care facilities, I.R.Iran, 2001-2011:**
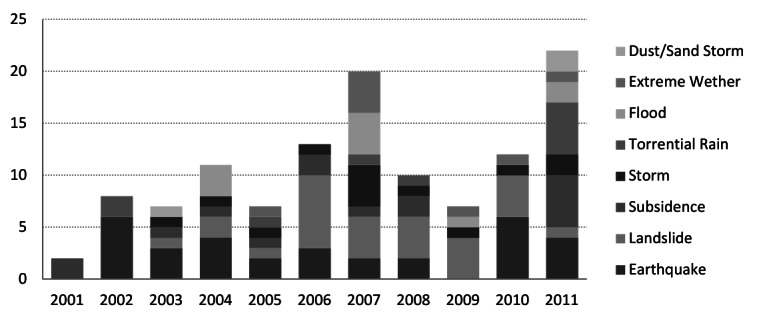

While there is variability from year to year, the overall trend over the last decade demonstrated almost 20% increase in such impacts each year (figure 4). The adverse impacts on health centers included structural damage (697 centers, 49.8%), non-structural damage (842 centers, 60.1%) and functional failure (1,378 centers, 98.4%) with a large proportion of those impacts 40.6%, 56.6% and 91.9% respectively, classified as moderate to severe. The specific impacts of each type of natural hazard event are characterized in figure 5 demonstrating the extensive impact of hydro-meteorological events, especially on non-structural and functional elements.

**Number of damaged primary health care facility by natural hazards, I.R.Iran, 2001-2011:**
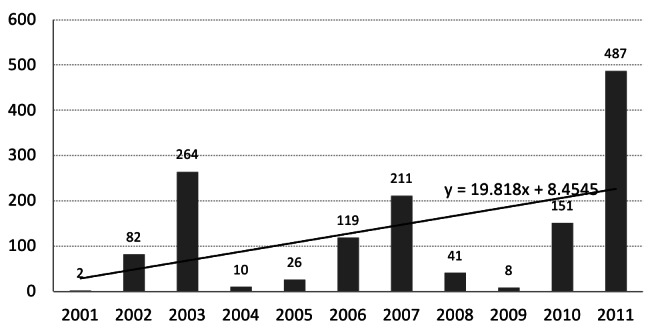

**Severity of natural hazards impact on primary health care facilities by types of hazard and damage, I.R.Iran, 2001-2011:**
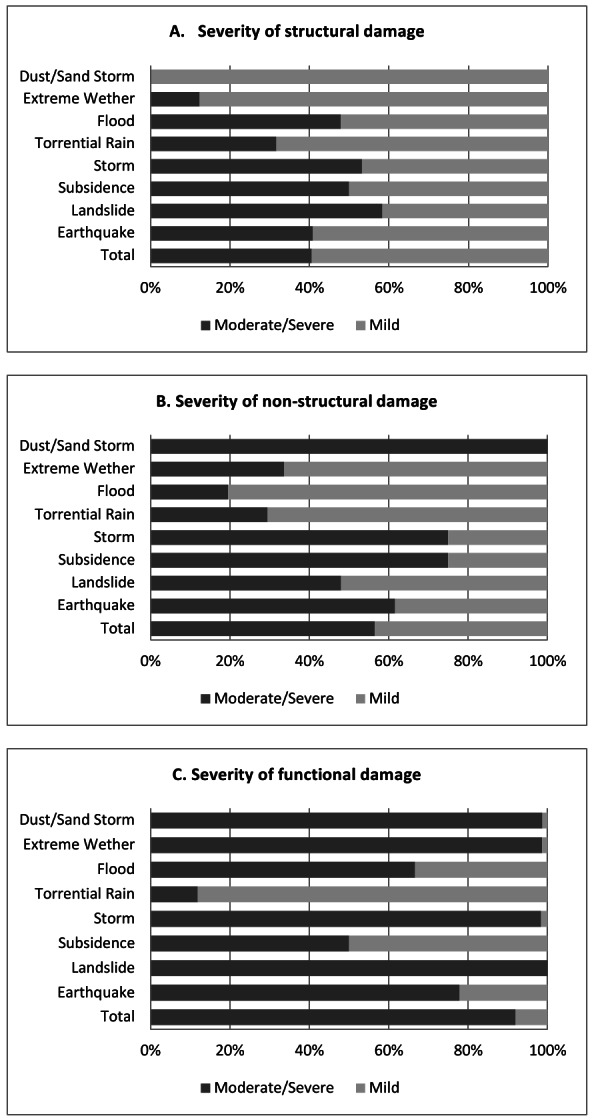

Table 2 presents the crude and adjusted number of affected health centers by their type. Adjusted for the total number of centers, the most affected centers were rural health centers (RHC) followed by urban health centers (UHC) in terms of structural damage. Urban health centers suffered the most non-structural damage followed by RHCs and district public health centers. Urban health centers followed by health houses and RHCs suffered the most functional failure due to natural hazards.

**Table 2: Number of affected primary health care centers due to natural hazards by types of facility and damage, I.R.Iran, 2001-2011:** *Based on average number of centers in 2001 and 2011.

Facility type	Number of centers	Structural damage		Non-Structural damage		Functional failure	
		N	%	N	%	N	%
Health house	13,638	440	3.2	553	4.1	1,057	7.8
Rural health center	1,763	146	8.3	116	6.6	115	6.5
Health post	1,364	42	3.1	32	2.3	78	5.7
Urban health center	795	55	6.9	119	15.0	115	14.5
District public health center	287	8	2.8	17	5.9	5	1.7
District health network center	287	6	2.1	5	1.7	8	2.8
**Total**	**18,134**	**697**	**3.8**	**842**	**4.6**	**1,378**	**7.6**

Using a threshold of 50 or more deaths and/or 500 buildings damaged 9, four hazardous events were categorized as *intensive *risk including the Bam earthquake (2003), Zarand earthquake (2005), Lorestan earthquake (2006) and Gonu cyclone (2007). These four events represented only 3.4% of all hazards measured over this decade yet accounted for damage to 374 health centers (27.6%). Hazards with *extensive *risk (with less than 50 deaths and/or 500 buildings damaged) were in turn responsible for moderate to severe damage to PHC facilities in 25.6%, 27.9% and 58.2% for structural, non-structural and functional failure, respectively (figure 6).

**Moderate and severe impacts of natural hazards on primary health care facilities: Intensive vs. extensive , I.R.Iran, 2001-2011:**
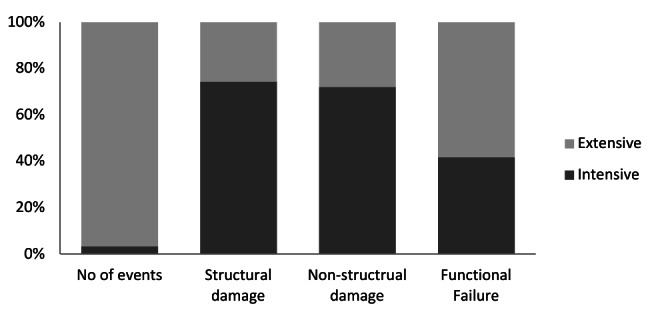

## Discussion

This study was the first of its kind in I.R.Iran and one of the first globally to specifically analyze the impacts of natural hazards on primary health care facilities. The data demonstrated that on average more than 140 primary health centers were affected by natural disasters each year. Both the incidence of natural hazard events as well as their impacts on primary health centers have increased annually.

Earthquakes were the cause of all death among health personnel and the most destructive hazard in term of structural and non-structural damages to primary health facilities. While geologic hazards significantly hampered health service delivery, hydro-meteorological hazards were also responsible for one third of functional failures. The role of extensive hazards in ¼ of physical damage and 2/3 of functional failure is also worthy of mention. In terms of crude number, health houses were the most affected, but a higher rate of damage was found in rural and urban health centers.

This study’s primary limitation is that it is a retrospective review. There was no registry of impacts of natural hazard events on PHCs in Iran over the study period. Furthermore, since the data that was being collected was collateral information originally collected for other purposes (e.g. maintenance logs) it is possible that some impacts were not recorded. The lack of a registry of such impacts made this limitation unavoidable. With the inclusion of 25 out of 30 provinces and 36 of 42 public health departments, however, it is expected that this study captured the overwhelming majority if not all of the important hazardous events.

A further limitation of this retrospective study was the necessary reliance on contemporaneously recorded archival records at each PHD as well as key informant interviews conducted often several years after the events took place. While steps were taken to corroborate data collected from multiple sources, the dataset is inevitably subject to some bias based on the variable quality of data in each PHD. While we could not fully control for this variability, based on the characteristics of responding and non-responding departments with respect to the number of PHCs represented and natural hazards occurring we have no reason to suspect a non-random rate of data loss.

With the increased focus on the impacts of natural disasters in the I.R.Iran, there is undoubtedly an observation bias with some of the increased incidence of these events being accounted for by better recording of the events and their impacts. However, the trend parallels the increased incidence of other more robustly collected measures such as incidence of hydro-meteorological hazards over the last two decades in Iran 8. These findings should be interpreted carefully in term of innate limitation of such a retrospective review but they indicate the real possibility of significant and worsening impacts of natural disasters on PHC facilities. Additionally, there is a recall bias associate with recalling remote events (up to ten years prior). We feel that this bias minimally impact the data as it is only relevant for data obtained through key informant interviews for which no corroborating documents could be found. Further prospective study of these phenomena is needed to more accurately describe the scale and scope of these impacts.

It was an expected result that earthquakes were the main source of death and injury and with significant damage to PHC facilities. The unexpected finding in these data was the extent to which hydro-meteorological hazards impacted these centers. Most of these were related to extreme weather events and dust/sand storms. Iran has been faced with repeated occurrence of dust/sand storms in south and west provinces that originated from Iraq 10. While these storms did not lead to physical damage the resultant air pollution caused frequent illnesses of health staff and closing the health centers when pollution levels exceeded a defined threshold.

Keeping the importance of intensive risks events in mind, the impact of extensive risks hazards, on health system must be underlined in disaster mitigation and preparedness planning. According to definition provided by GAR 2009 9 for the intensive risks, i.e. hazards with more than 50 deaths and 500 buildings damaged, we recorded four disasters in this category including Bam (2003), Zarand (2005), Lorestan (2006) earthquakes and cyclone Gonu (2007). While the impacts of these hazards were well documented because of the intense media attention they received, this study demonstrated that, in fact, 2/3 of functional failure and ¼ of physical damage of primary health centers occurred from *extensive *not *intensive *hazards. This corresponds with previous findings in GAR 2009 that 2/3 of damages to hospitals over two decades were due to extensive risks 9.

It was noted in the course of this study that the same structural design plan was used for all primary health centers around the country irrespective of the hazard vulnerability profile of each health center site. This undoubtedly contributed to excess impacts due to natural hazards. The land use planning and design methods should be adapted to the vulnerability of each area 2. We propose that a set of different design types should be created for implementation in different parts of the country to mitigate against the disaster types that are most prevalent in each area. This is a shared responsibility of health planners, engineers, architects and administrators.

While hospitals and primary health care centers are components of the same system and have interrelated functions, there are functions that are specific to primary health centers that cannot be replaced by hospitals. These functions are community disaster preparedness before a disaster and providing public health services afterwards. Expanded geographical distribution of primary centers all over the country and easy access of people even in remote areas are great advantage of the PHC centers and make their preservation important in times of crisis. Furthermore, a resilient and functional primary health center will minimize the number of non-seriously injured people who might approach hospitals for care. The designs of PHCs are less complex and are less costly to retrofit to improve resilience to natural hazards.

Iran has previous experience with rapid visual screening of 540 public hospitals for earthquake resistance in 2010 that led to a 10-year plan ratified by Iran’s Islamic Parliament 11. Since early 2012, Iran’s health system has focused on development of disaster risk reduction (DRR) programs in PHC network. The highlights of these efforts are developing a program for integration of DRR in PHC network and developing tools for hazard and vulnerability assessment in PHC facilities 12. In addition, a steering committee has been shaped to specifically address the physical vulnerability of PHC facilities against disasters. As a result, the 2012-2013 operational plan of Iran’s Ministry of Health & Medical Education included the hazard and vulnerability assessment (HVA) of PHC centers.

Despite high exposure and vulnerability of primary health facilities to natural hazards, at the time of the study most of the PHCs were not covered by any insurance plan. Fortunately, since early 2012, all public buildings including health system facilities have been required by law to have such a plan.

## Conclusion

Iran’s PHC facilities have been increasingly impacted by such events during the last decade. As a result of this study, starting in 2012 all PHCs have been mandated to report any hazardous event that impacts health facilities and resources and the type of impacts sustained. To reduce the impact of disasters on PHC facilities, Iran’s health system needs to focus on: hazard and vulnerability assessment of existing centers; cost-benefit analysis of retrofitting vulnerable centers; enforcing comprehensive disaster mitigation standards in construction of new facilities; emphasizing hazard-specific emergency plans especially for storms, floods and dust/sand storm using an all-hazard approach.

To make all these happen Iran’s health system needs to continue the integration of disaster risk reduction into PHC public health programs. As a very basic step, indicators related to hazard and vulnerability assessment of PHC facilities should be included in National Health Information System. This initiative will equip the health system with baseline and trend data regarding the HVA of the facilities for policy making and effective resource allocation.

## Competing Interests

The authors have declared that no competing interests exist.

## Appendix 1

### Data Collection Form

Download PDF
